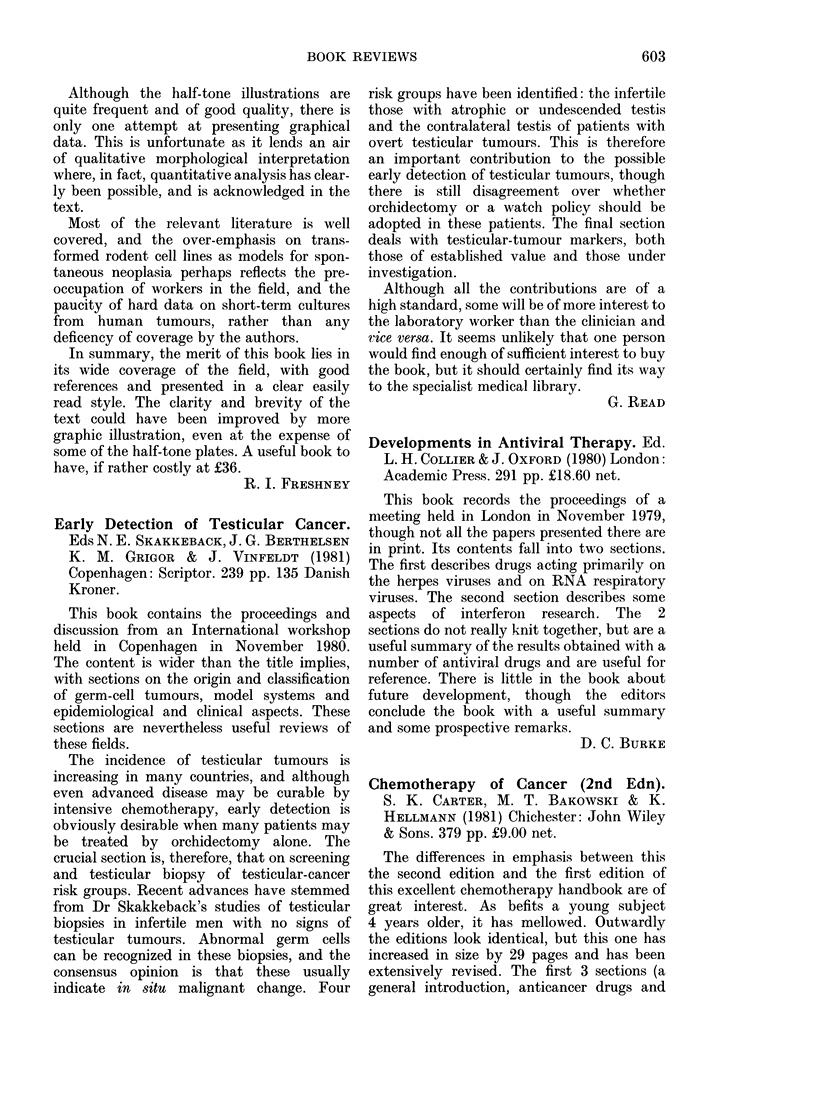# Early Detection of Testicular Cancer

**Published:** 1981-10

**Authors:** G. Read


**Early Detection of Testicular Cancer.** Eds N. E. Skakkeback, J. G. Berthelsen K. M. Grigor & J. Vinfeldt (1981) Copenhagen: Scriptor. 239 pp. 135 Danish Kroner.

This book contains the proceedings and discussion from an International workshop held in Copenhagen in November 1980. The content is wider than the title implies, with sections on the origin and classification of germ-cell tumours, model systems and epidemiological and clinical aspects. These sections are nevertheless useful reviews of these fields.

The incidence of testicular tumours is increasing in many countries, and although even advanced disease may be curable by intensive chemotherapy, early detection is obviously desirable when many patients may be treated by orchidectomy alone. The crucial section is, therefore, that on screening and testicular biopsy of testicular-cancer risk groups. Recent advances have stemmed from Dr Skakkeback's studies of testicular biopsies in infertile men with no signs of testicular tumours. Abnormal germ cells can be recognized in these biopsies, and the consensus opinion is that these usually indicate *in situ* malignant change. Four risk groups have been identified: the infertile those with atrophic or undescended testis and the contralateral testis of patients with overt testicular tumours. This is therefore an important contribution to the possible early detection of testicular tumours, though there is still disagreement over whether orchidectomy or a watch policy should be adopted in these patients. The final section deals with testicular-tumour markers, both those of established value and those under investigation.

Although all the contributions are of a high standard, some will be of more interest to the laboratory worker than the clinician and *vice versa*. It seems unlikely that one person would find enough of sufficient interest to buy the book, but it should certainly find its way to the specialist medical library.

G. Read